# Clinical and Neuropathological Correlates of the *APOE* Genotype in Frontotemporal Lobar Degeneration‐Tau (FTLD‐Tau)

**DOI:** 10.1002/alz70855_106234

**Published:** 2025-12-24

**Authors:** Jing Qian, Weidong Wang, Bradley T. Hyman, Alberto Serrano‐Pozo

**Affiliations:** ^1^ University of Massachusetts, Amherst, MA, USA; ^2^ Massachusetts Alzheimer's Disease Research Center, Charlestown, MA, USA; ^3^ Harvard Medical School, Boston, MA, USA; ^4^ Massachusetts General Hospital, Boston, MA, USA

## Abstract

**Background:**

The *APOE* genotype is the strongest genetic modulator of Alzheimer's disease (AD), affecting not only the risk of developing AD, but also its age of onset and neuropathological features, particularly amyloid plaque burden and cerebral amyloid angiopathy (CAA) severity. In contrast, the relationships between the *APOE* genotype and both antemortem clinical outcomes and postmortem neuropathological findings in the context of FTLD‐Tau are less well‐explored, despite their potentially important prognostic and therapeutic implications.

**Method:**

We queried the National Alzheimer's Coordinating Center (NACC) database (September 2024 data freeze). Eligibility criteria were: (1) brain autopsy available; (2) *APOE* genotype available and not ε2/ε4; (3) last clinical evaluation within 2 years from death including clinical dementia rating sum of boxes (CDR‐SOB) score; and (4) a neuropathological diagnosis of FTLD‐Tau (regardless of comorbid neuropathologies). Statistical analyses included Cox proportional hazards, multivariable linear and logistic regression as well as mediation models. Age at death, sex, and education were adjusted for whenever appropriate.

**Result:**

A total of 764 subjects met eligibility criteria, including 108 *APOE*ε2 carriers, 466 *APOE*ε3 homozygotes, and 190 *APOE*ε4 carriers (173 *APOE*ε3/ε4 and 17 *APOE*ε4 homozygotes). Regarding clinical correlates, relative to the *APOE*ε3/ε3 genotype (reference group), *APOE*ε4 carriage was associated with a significantly younger age of onset and higher antemortem CDR‐SOB score, whereas *APOE*ε2 carriage did not impact age of onset and showed a non‐significant trend toward a lower CDR‐SOB score. Regarding neuropathological correlates, carrying the *APOE*ε4 allele was associated with higher CERAD neuritic plaque score and Braak NFT stage, more severe CAA and arteriolosclerosis, and the presence of Lewy bodies, whereas carrying the *APOE*ε2 allele was associated with a lower CERAD neuritic plaque score. Of all comorbid neuropathologies analyzed, only neuritic plaques, arteriolosclerosis, and hippocampal sclerosis were independently associated with a higher antemortem CDR‐SOB score. Mediation analyses revealed that the effects of both *APOE*ε2 and *APOE*ε4 alleles on antemortem CDR‐SOB score are largely indirect, i.e. mediated by their effects on neuritic plaques, and in the case of *APOE*ε4, also on arteriolosclerosis.

**Conclusion:**

The *APOE* genotype impacts clinical outcomes in FTLD‐Tau, but largely through its modulation of AD and other comorbid pathologies.

